# Navigating Evidence, Challenges, and Caution in the Treatment of Stimulant Use Disorders

**DOI:** 10.3390/brainsci13101416

**Published:** 2023-10-06

**Authors:** Anees Bahji, Marlon Danilewitz, David Crockford

**Affiliations:** 1 Department of Psychiatry, University of Calgary, Calgary, AB T2R 0N2, Canada; 2 Department of Community Health Sciences, University of Calgary, Calgary, AB T2R 0N2, Canada; 3 Hotchkiss Brain Institute, University of Calgary, Calgary, AB T2R 0N2, Canada; 4 Department of Psychiatry, University of Toronto, Toronto, ON M5T 1R7, Canada; danilewitzm@ontarioshores.ca; 5 Ontario Shores Center for Mental Health Sciences, Whitby, ON L1N 5S9, Canada

**Keywords:** stimulant, addiction

## Abstract

Amidst the opioid epidemic, harm reduction-oriented approaches have gained traction, including interventions that focus on prescribing pharmaceutical-grade psychoactive substances, such as opioids, instead of illicit versions, intending to mitigate fatal overdose risks arising from the variability in potency and additives found in illicit drugs. Stimulants have increasingly been found in the victims of opioid overdoses, further prompting some to argue for the prescription of stimulant medications for individuals with stimulant use disorders. Yet, the evidence supporting this practice remains insufficient. In this communication, we critically examine the existing evidence, challenges, and cautions surrounding the treatment of stimulant use disorder.

## 1. Introduction

Stimulants have well-known short-term euphoric effects, including wakefulness, appetite suppression, and heightened attention, causing them to be sought out and used illegally on a global basis [1]. Cocaine and methamphetamine are increasingly reported as being present in drug toxicity deaths related to opioids [2]. People who use stimulants are less likely to be retained on opioid agonist therapy (OAT), further increasing their risk of a potentially fatal overdose [2]. Stimulants are often used with opioids for various reasons, including the promotion of alertness and wakefulness, especially in homeless people, to reduce the likelihood of theft, assault, or other forms of victimization. Stimulants can also be used to prolong the effects of opioids [3], contribute to high-risk sexual activity [4], and can be taken based on social influences [5,6,7] or under the mistaken belief that using a stimulant with an opioid may reduce the risk of overdose, even though the data points to the contrary [2,8,9,10,11].

The concomitant use of stimulants and opioids and the relative success of OAT have prompted some authors to argue for the use of prescription stimulants as a form of safe supply for individuals with stimulant use disorders, with or without opioid use disorders. While these efforts aim to mitigate harm and decrease mortality, there is a noted lack of robust evidence supporting the effectiveness and safety of the prescription of stimulants for individuals with stimulant use disorders. Despite decades of research, there are currently no approved medications for treating stimulant use disorder [12]. While a meta-analysis conducted by Tardelli et al. in 2020 yielded positive findings—stating that prescription amphetamines have a beneficial effect for promoting abstinence in individuals with cocaine use disorder [13]—most reviews and meta-analyses have consistently highlighted the low quality of evidence, high dropout rates, exclusion of common comorbidities, and potential adverse reactions and side effects from high-dose psychostimulant exposure [14,15,16,17,18].

## 2. The Complex Nature of Stimulant Use Disorder

Stimulant use disorders present unique challenges and are usually characterized by complex antecedents and consequences from use that would not respond to pharmacological approaches alone [19]. As described above, individuals who use stimulants do so for various reasons and have complicated medical, social, and psychological needs. Medical comorbidity is common, involving neurologic injuries, viral hepatitis, HIV, and other medical problems [1]. Moreover, psychiatric comorbidity is also commonplace, involving trauma, PTSD, psychosis, and depression (reference as above). Engagement with this population can also be challenging, given multiple barriers. 

The complex situation involving providing treatment for people with stimulant use disorders thus requires extensive wrap-around services to help manage work pertaining to engagement, trust building, and providing meaningful improvements to their quality of life. Wrap-around services should ideally co-occur, addressing housing, financial, social, medical, and psychiatric needs. All too often, though, when people seek help, they encounter delays, get care that only focuses on one or a few aspects of their problems, or do not receive any care. While there is often debate about potential elements of care, like safe supply, that are potentially easier to implement as all they require is a prescription, they generally come at the expense of ignoring the larger problem, which can seem more overwhelming. Ultimately, the complex nature of stimulant use disorders necessitates an equally complex and comprehensive approach to reduce the potential for overdose and improve overall outcomes effectively [19].

## 3. Potential Risks of Prescribed Stimulants

Given the lack of robust evidence for its efficacy, expanding stimulant prescriptions for individuals with stimulant use disorder raises serious concerns [20,21]. These individuals commonly experience symptoms of psychosis and agitation, which can be exacerbated by prescription stimulants, even when used alongside antipsychotic medications [22]. For example, amongst individuals using methamphetamines, the risk of developing psychosis is profound, with estimates that 30–40% of persons using methamphetamine develop psychosis [23,24]. Once psychosis has developed, it is prone to recurrence and worsening with exposure to stimulants [23]. Any off-label prescription stimulants for stimulant use disorders would require screening for the presence of psychosis and avoiding use in this population.

The use of methamphetamine and cocaine is associated with violence, accidental injuries, and homicide [1,25]. It is unclear if the prescription of stimulants would reduce this risk, particularly for those who have experienced psychosis or continue to use non-prescribed substances. 

Stimulants also have known detrimental cardiovascular effects, including hypertension, electrocardiogram (ECG) abnormalities, myocardial infarction, and stroke [1]. Cardiovascular monitoring would need to be part of prescribing, especially if people are also being prescribed or using agents known to cause ECG abnormalities or if they have known cardiovascular disease.

Further, prescription stimulants are prone to diversion and misuse, especially in persons with substance use disorders [25]. As prescription stimulants are full dopamine receptor agonists, they do not limit—and instead enhance—the effects of illicit stimulant co-use. Only extended-release preparations should be used, rather than short-acting preparations, to determine subjective euphoric effects. Prescription stimulants would require close medical monitoring, with daily, supervised administration warranted to allow for the ongoing evaluation and mitigation of safety risks. In addition, the continuous weighing of potential risks associated with prescriptions balanced against any potential benefits needs to be conducted.

## 4. Challenges in Supporting Prescribed Stimulants

Presently, there is a lack of robust evidence to support the widespread integration of prescription stimulants in the treatment of stimulant or other substance use disorders [10]. Unlike substitution therapies for opioid use disorders, the evidence supporting substitution therapy for stimulant use disorders is not clear. While numerous reviews have explored the use of prescribed psychostimulants for stimulant use disorders, it is essential to note that only one meta-analysis conducted by Tardelli et al. (2020) reached a positive conclusion [13,26]. Most other reviews and meta-analyses have consistently highlighted the lack of evidence supporting pharmacological interventions for these disorders [12,14,27,28]. They have emphasized the low-grade quality of the evidence, high dropout rates potentially leading to bias, the absence of improvements in treatment retention, the exclusion of common comorbidities, and potential adverse reactions and side effects from high-dose psychostimulant exposure [14,15,16]. For example, Siefried et al., 2020 [29] and Lee et al., 2018 [30] concluded that no pharmacotherapy yielded convincing results for the treatment of amphetamine or methamphetamine dependence.

Furthermore, while substitution therapy with opioid agonists has shown reductions in overdoses and mortality for opioid use disorders [31], it is worth noting that one observational study suggested the use of lisdexamfetamine improved outcomes for individuals with amphetamine or methamphetamine use disorders [32]. However, it is unknown in that study how much was prescribed for attention deficit hyperactivity disorder or off-label for stimulant use disorder. Nevertheless, the current evidence supporting the efficacy of prescribed stimulants in treating stimulant use disorders remains limited [2,20,33].

## 5. ADHD and Stimulant Use Disorder

Attention deficit hyperactivity disorder (ADHD) appears to be common in persons with stimulant use disorders [34,35]. However, diagnosis of ADHD in persons with stimulant use disorders can be challenging as both intoxication and withdrawal from stimulants can mimic ADHD symptoms; intoxication produces hyperactivity and impulsivity, and withdrawal produces impairments in attention, concentration, and working memory, with distractibility nearly indistinguishable from ADHD that can linger for weeks to months [34,35]. Symptomatic screening instruments like the Adult ADHD Self-Report Scale Short Version (ASRS) have marked limitations in aiding diagnosis for this patient group. As such, most clinicians recommend delaying diagnosis for weeks to months [36,37], trying to establish a clearer history of the onset of symptoms prior to the age of 12 before considering there to be a diagnosis of ADHD present [34].

Even when a diagnosis of ADHD seems likely, most clinicians remain reluctant to prescribe psychostimulants to patients with stimulant use disorders due to the potential risk of worsening stimulant use disorder outcomes, misuse, diversion, or the use of such medications limiting access to addiction treatment programs [37,38]. To this end, most guidance recommends using a non-stimulant medication, like atomoxetine, for persons with stimulant use disorder history first-line rather than a long-acting prescription stimulant, even though effect sizes are larger for the long-acting prescription stimulants for established ADHD [39,40,41].

However, some have suggested that tolerance to stimulants necessitates higher doses of prescribed psychostimulants to be effective [42,43]. Two RCTs have demonstrated improvements in ADHD and SUD (amphetamine and cocaine) with higher doses of long-acting methylphenidate (180 mg/day) [44] or sustained-release mixed amphetamine salts (60–80 mg/day) [45]. Still, the former study reported extremely high dropout rates (19/27 in the treatment group and 25/27 in the placebo group), and the latter study found no differences between 60 mg and 80 mg dosing or improvements in treatment retention over placebo; thus, the data is only preliminary [34].

Overall, then, there may be a subset of persons with stimulant use disorders who clearly have ADHD and may benefit from a prescription stimulant. Still, they are challenging to diagnose accurately, and it remains unclear if the long-acting prescription stimulants would be the best medication to use in this group [34].

## 6. Effect on Cravings for Illicit Stimulants

Clinical experience has shown that prescribed stimulants can paradoxically increase cravings for stimulants, potentially reactivating drug craving, seeking, and use due to shared neurobiological pathways [46]. Thus, another critical consideration is the potential impact of prescribed stimulants on cravings for illicit stimulants. While it is postulated that prescription stimulants may help reduce cravings, the evidence remains inconclusive [17,18,29,47]. Neurobiological evidence indicates that using drugs of a similar class or activating shared pathways can reinstate drug craving, seeking, and use [46]. Therefore, it is essential to carefully assess how prescribed stimulants interact with the existing neural pathways and behaviours related to stimulant use disorder.

## 7. Psychosocial Interventions: An Evidence-Based Approach

Psychosocial interventions have more established efficacy for treating stimulant use disorders than pharmacotherapy options. These interventions encompass a range of therapeutic approaches that address addiction’s psychological, behavioural, and social aspects. Psychosocial interventions help individuals develop coping skills, enhance motivation for change, improve treatment adherence, and reduce the risk of relapse [48]. Incorporating psychosocial interventions into the treatment approach for stimulant use disorder also acknowledges the multifaceted nature of addiction while recognizing that successful treatment requires a holistic approach that encompasses various aspects of an individual’s life [49].

One prominent example is contingency management (CM). This widely studied and effective psychosocial intervention provides tangible rewards or incentives to individuals when they achieve specific treatment goals, such as abstinence from stimulant use or adherence to treatment plans. In the context of stimulant use disorders, CM has demonstrated significant efficacy in increasing the likelihood of negative cocaine test results among adults and helping promote and strengthen desirable behaviours while reducing substance use [50,51,52,53,54].

Beyond CM, other psychosocial interventions have also shown promise in treating stimulant use disorder. For example, cognitive behavioural therapy (CBT) is a well-established approach that focuses on identifying and modifying maladaptive thoughts, beliefs, and behaviours associated with substance use. CBT has been shown to effectively reduce stimulant use and improve treatment outcomes by addressing cognitive distortions, developing coping skills, and enhancing motivation for change [15,55,55,56,57,58].

Motivational interviewing (MI) is another valuable psychosocial intervention to enhance intrinsic motivation and facilitate behavioural change. Through empathetic and collaborative conversations, MI helps individuals explore and resolve their ambivalence about substance use, increase their readiness for change, and develop personalized goals for recovery. Several robust studies demonstrate the evidence of MI-based interventions to help with substance use disorders, including stimulant use disorders [59,60,61,62].

Family-based interventions are crucial in addressing the social context of stimulant use disorders [63]. These interventions involve the participation of family members in the treatment process, focusing on improving family dynamics, communication, and support [64]. By involving the family system, these interventions can enhance the individual’s social support network, reinforce positive behaviours, and facilitate recovery [65].

Group therapy and support groups, such as 12-step programs like Narcotics Anonymous (NA) and SMART Recovery, allow individuals with stimulant use disorder to connect with peers who share similar experiences [66]. These group-based interventions offer a supportive and non-judgmental environment where individuals can gain insight, share strategies for coping with cravings and triggers, and receive encouragement from others in recovery.

Recent comprehensive reviews, including a systematic review [67] and a network meta-analysis [68], have synthesized the findings from over 50 randomized controlled trials, examining the effectiveness and acceptability of various psychosocial interventions tailored specifically for stimulant use disorders, encompassing both cocaine and amphetamine use disorders. These reviews collectively underscore the presence of robust evidence for a range of psychosocial interventions, indicating both efficacy (measured by abstinence rates) and acceptability (characterized by retention in treatment) for a diverse set of 12 psychosocial interventions when compared to standard treatment protocols. These interventions encompass contingency management, the community reinforcement approach, CBT, 12-step programs, meditation-based approaches, physical exercise regimens, supportive-expressive psychodynamic therapy, interpersonal psychotherapy, family therapy, motivational interviewing, drug counselling, or their combinations.

## 8. Comprehensive Treatment Approach

Given these considerations, it is essential to prioritize evidence-based recovery treatments, housing, and comprehensive support services that address the complex needs of individuals with stimulant use disorder. Emphasizing the best available evidence and the need to improve psychosocial treatment offerings can provide valuable insights and guidance for addressing this specific issue. To that end, a robust wrap-around approach that integrates housing, medical care, psychiatric support provisions, and evidence-based psychosocial interventions should be universally implemented.

## 9. The Need for Objective Evaluation

While psychosocial interventions have shown effectiveness, further research is needed to enhance the understanding and treatment of stimulant use disorder. Well-designed clinical trials, even on a smaller scale, will provide valuable insights into the benefits and risks of prescribing different pharmacotherapeutic interventions, enabling informed decisions, and ensuring the safety and well-being of individuals with severe stimulant use disorder. By emphasizing the need for caution, evidence-based approaches, and well-designed clinical trials, we can contribute to a more responsible and effective response to substance use disorders and ensure the best possible outcomes for individuals seeking treatment. In concert, there is a need for objective clinical trials involving patients commonly encountered in clinical practice, which should be conducted in order to assess critical factors such as adherence, changes in substance use patterns, retention in care, cravings for drug use, and incidences of overdoses.

## References

[1] Farrell M., Martin N.K., Stockings E., Bórquez A., Cepeda J.A., Degenhardt L., Ali R., Tran L.T., Rehm J., Torrens M. (2019). Responding to Global Stimulant Use: Challenges and Opportunities. Lancet.

[2] Mintz C.M., Xu K.Y., Presnall N.J., Hartz S.M., Levin F.R., Scherrer J.F., Bierut L.J., Grucza R.A. (2022). Analysis of Stimulant Prescriptions and Drug-Related Poisoning Risk Among Persons Receiving Buprenorphine Treatment for Opioid Use Disorder. JAMA Netw. Open..

[3] Compton W.M., Valentino R.J., DuPont R.L. (2021). Polysubstance Use in the U.S. Opioid Crisis. Mol. Psychiatry.

[4] Moradi S., Moradi Y., Rahmani K., Nouri B., Moradi G. (2022). The Association between Methamphetamine Use and Number of Sexual Partners in Men Who Have Sex with Men: A Systematic Review and Meta-Analysis. Subst. Abus. Treat. Prev. Policy.

[5] Nyamathi A., Hudson A., Greengold B., Leake B. (2012). Characteristics of Homeless Youth Who Use Cocaine and Methamphetamine. Am. J. Addict..

[6] Carrillo Beck R., Szlapinski J., Pacheco N., Sabri Laghaei S., Isard R., Oudshoorn A., Marshall C.A. (2022). Violence and Victimisation in the Lives of Persons Experiencing Homelessness Who Use Methamphetamine: A Scoping Review. Health Soc. Care Community.

[7] Homer B.D., Solomon T.M., Moeller R.W., Mascia A., DeRaleau L., Halkitis P.N. (2008). Methamphetamine Abuse and Impairment of Social Functioning: A Review of the Underlying Neurophysiological Causes and Behavioral Implications. Psychol. Bull..

[8] Palis H., Gan W., Xavier C., Desai R., Scow M., Sedgemore K., Greiner L., Nicholls T., Slaunwhite A. (2022). Association of Opioid and Stimulant Use Disorder Diagnoses With Fatal and Nonfatal Overdose Among People With a History of Incarceration. JAMA Netw. Open..

[9] Ahmed S., Sarfraz Z., Sarfraz A. (2022). Editorial: A Changing Epidemic and the Rise of Opioid-Stimulant Co-Use. Front. Psychiatry.

[10] Palis H., Xavier C., Dobrer S., Desai R., Sedgemore K., Scow M., Lock K., Gan W., Slaunwhite A. (2022). Concurrent Use of Opioids and Stimulants and Risk of Fatal Overdose: A Cohort Study. BMC Public. Health.

[11] Ciccarone D. (2021). The Rise of Illicit Fentanyls, Stimulants and the Fourth Wave of the Opioid Overdose Crisis. Curr. Opin. Psychiatry.

[12] Haile C.N., Kosten T.R. (2013). Pharmacotherapy for Stimulant-Related Disorders. Curr. Psychiatry Rep..

[13] Tardelli V.S., Bisaga A., Arcadepani F.B., Gerra G., Levin F.R., Fidalgo T.M. (2020). Prescription Psychostimulants for the Treatment of Stimulant Use Disorder: A Systematic Review and Meta-Analysis. Psychopharmacology.

[14] Chan B., Kondo K., Freeman M., Ayers C., Montgomery J., Kansagara D. (2019). Pharmacotherapy for Cocaine Use Disorder—A Systematic Review and Meta-Analysis. J. Gen. Intern. Med..

[15] Ronsley C., Nolan S., Knight R., Hayashi K., Klimas J., Walley A., Wood E., Fairbairn N. (2020). Treatment of Stimulant Use Disorder: A Systematic Review of Reviews. PLoS ONE.

[16] de Lima M.S., de Soares B.G.O., Reisser A.A.P., Farrell M. (2002). Pharmacological Treatment of Cocaine Dependence: A Systematic Review. Addiction.

[17] Khoramizadeh M., Effatpanah M., Mostaghimi A., Rezaei M., Mahjoub A., Shishehgar S. (2019). Treatment of Amphetamine Abuse/Use Disorder: A Systematic Review of a Recent Health Concern. DARU J. Pharm. Sci..

[18] Shoptaw S.J., Kao U., Heinzerling K., Ling W. (2009). Treatment for Amphetamine Withdrawal. Cochrane Database Syst. Rev..

[19] Halkitis P.N. (2009). Motivations and Antecedents of Methamphetamine Use: Risk Bases. Methamphetamine Addiction: Biological Foundations, Psychological Factors, and Social Consequences.

[20] Westover A.N., Nakonezny P.A., Halm E.A., Adinoff B. (2018). Risk of Amphetamine Use Disorder and Mortality among Incident Users of Prescribed Stimulant Medications in the Veterans Administration. Addiction.

[21] Compton W.M., Han B., Blanco C., Johnson K., Jones C.M. (2018). Prevalence and Correlates of Prescription Stimulant Use, Misuse, Use Disorders, and Motivations for Misuse Among Adults in the United States. AJP.

[22] Sommers I., Baskin D. (2006). Methamphetamine Use and Violence. J. Drug. Issues.

[23] Crockford D.N., Meunier S., Ghosh S.M. (2019). Methamphetamine-Induced Psychosis: A Clinician’s Guide. Can. J. Addict..

[24] Glasner-Edwards S., Mooney L.J. (2014). Methamphetamine Psychosis: Epidemiology and Management. CNS Drugs.

[25] United Nations Office on Drugs and Crime (2019). Treatment of Stimulant Use Disorders: Current Practices and Promising Perspectives.

[26] Bahji A., Dhaliwal A., Sachdev A., Danilewitz M., Lamba W., George T.P., Chopra N., Crockford D. (2023). The Rising Tide of Stimulant-Related Morbidity and Mortality Warrants Evidence-Based Treatment. Can. J. Psychiatry.

[27] Brandt L., Chao T., Comer S.D., Levin F.R. (2021). Pharmacotherapeutic Strategies for Treating Cocaine Use Disorder—What Do We Have to Offer?. Addiction.

[28] Liu M.T. (2021). Pharmacotherapy Treatment of Stimulant Use Disorder. Ment. Health Clin..

[29] Siefried K.J., Acheson L.S., Lintzeris N., Ezard N. (2020). Pharmacological Treatment of Methamphetamine/Amphetamine Dependence: A Systematic Review. CNS Drugs.

[30] Lee N.K., Jenner L., Harney A., Cameron J. (2018). Pharmacotherapy for Amphetamine Dependence: A Systematic Review. Drug. Alcohol. Depend..

[31] Bahji A., Cheng B., Gray S., Stuart H. (2019). Reduction in Mortality Risk with Opioid Agonist Therapy: A Systematic Review and Meta-Analysis. Acta Psychiatr. Scand..

[32] Heikkinen M., Taipale H., Tanskanen A., Mittendorfer-Rutz E., Lähteenvuo M., Tiihonen J. (2023). Association of Pharmacological Treatments and Hospitalization and Death in Individuals With Amphetamine Use Disorders in a Swedish Nationwide Cohort of 13 965 Patients. JAMA Psychiatry.

[33] Black J.C., Bau G.E., Iwanicki J.L., Dart R.C. (2021). Association of Medical Stimulants With Mortality in the US From 2010 to 2017. JAMA Intern. Med..

[34] Ward B., Bahji A., Crockford D. (2022). Managing Adult Attention-Deficit/Hyperactivity Disorder With Comorbid Substance Use Disorder. Can. J. Addict..

[35] Levin F.R. (2007). Diagnosing Attention-Deficit/Hyperactivity Disorder in Patients with Substance Use Disorders. J. Clin. Psychiatry.

[36] Katzman M.A., Bilkey T.S., Chokka P.R., Fallu A., Klassen L.J. (2017). Adult ADHD and Comorbid Disorders: Clinical Implications of a Dimensional Approach. BMC Psychiatry.

[37] Magon R., Müller U. (2012). ADHD with Comorbid Substance Use Disorder: Review of Treatment. Adv. Psychiatr. Treat..

[38] Kollins S.H. (2008). A Qualitative Review of Issues Arising in the Use of Psycho-Stimulant Medications in Patients with ADHD and Co-Morbid Substance Use Disorders. Curr. Med. Res. Opin..

[39] CADDRA (2018). Canadian ADHD Resource Alliance (CADDRA) Canadian ADHD Practice Guidelines.

[40] Kooij S.J., Bejerot S., Blackwell A., Caci H., Casas-Brugué M., Carpentier P.J., Edvinsson D., Fayyad J., Foeken K., Fitzgerald M. (2010). European Consensus Statement on Diagnosis and Treatment of Adult ADHD: The European Network Adult ADHD. BMC Psychiatry.

[41] Caye A., Swanson J.M., Coghill D., Rohde L.A. (2019). Treatment Strategies for ADHD: An Evidence-Based Guide to Select Optimal Treatment. Mol. Psychiatry.

[42] Iqbal M.N., Levin C.J., Levin F.R. (2019). Treatment for Substance Use Disorder With Co-Occurring Mental Illness. Focus. (Am. Psychiatr. Publ.).

[43] Crunelle C.L., van den Brink W., Moggi F., Konstenius M., Franck J., Levin F.R., van de Glind G., Demetrovics Z., Coetzee C., Luderer M. (2018). International Consensus Statement on Screening, Diagnosis and Treatment of Substance Use Disorder Patients with Comorbid Attention Deficit/Hyperactivity Disorder. Eur. Addict. Res..

[44] Konstenius M., Jayaram-Lindström N., Guterstam J., Beck O., Philips B., Franck J. (2014). Methylphenidate for Attention Deficit Hyperactivity Disorder and Drug Relapse in Criminal Offenders with Substance Dependence: A 24-Week Randomized Placebo-Controlled Trial. Addiction.

[45] Levin F.R., Mariani J.J., Specker S., Mooney M., Mahony A., Brooks D.J., Babb D., Bai Y., Eberly L.E., Nunes E.V. (2015). Extended-Release Mixed Amphetamine Salts vs Placebo for Comorbid Adult Attention-Deficit/Hyperactivity Disorder and Cocaine Use Disorder: A Randomized Clinical Trial. JAMA Psychiatry.

[46] Lecomte T., Dumais A., Dugré J.R., Potvin S. (2018). The Prevalence of Substance-Induced Psychotic Disorder in Methamphetamine Misusers: A Meta-Analysis. Psychiatry Res..

[47] Kampman K.M. (2008). The Search for Medications to Treat Stimulant Dependence. Addict. Sci. Clin. Pract..

[48] Jhanjee S. (2014). Evidence Based Psychosocial Interventions in Substance Use. Indian. J. Psychol. Med..

[49] Tran M.T.N., Luong Q.H., Le Minh G., Dunne M.P., Baker P. (2021). Psychosocial Interventions for Amphetamine Type Stimulant Use Disorder: An Overview of Systematic Reviews. Front. Psychiatry.

[50] Castells X., Cunill R., Pérez-Mañá C., Vidal X., Capellà D. (2016). Psychostimulant Drugs for Cocaine Dependence. Cochrane Database Syst. Rev..

[51] Bentzley B.S., Han S.S., Neuner S., Humphreys K., Kampman K.M., Halpern C.H. (2021). Comparison of Treatments for Cocaine Use Disorder Among Adults: A Systematic Review and Meta-Analysis. JAMA Netw. Open..

[52] Bolívar H.A., Klemperer E.M., Coleman S.R.M., DeSarno M., Skelly J.M., Higgins S.T. (2021). Contingency Management for Patients Receiving Medication for Opioid Use Disorder: A Systematic Review and Meta-Analysis. JAMA Psychiatry.

[53] Petry N.M. (2011). Contingency Management: What It Is and Why Psychiatrists Should Want to Use It. Psychiatrist.

[54] Rawson R.A., Erath T.G., Chalk M., Clark H.W., McDaid C., Wattenberg S.A., Roll J.M., McDonell M.G., Parent S., Freese T.E. (2023). Contingency Management for Stimulant Use Disorder: Progress, Challenges, and Recommendations. J. Ambul. Care Manag..

[55] Harada T., Tsutomi H., Mori R., Wilson D.B. (2018). Cognitive-Behavioural Treatment for Amphetamine-Type Stimulants (ATS)-Use Disorders. Cochrane Database Syst. Rev..

[56] AshaRani P., Hombali A., Seow E., Ong W.J., Tan J.H., Subramaniam M. (2020). Non-Pharmacological Interventions for Methamphetamine Use Disorder: A Systematic Review. Drug. Alcohol. Depend..

[57] McHugh R.K., Hearon B.A., Otto M.W. (2010). Cognitive-Behavioral Therapy for Substance Use Disorders. Psychiatr. Clin. N. Am..

[58] Smout M.F., Longo M., Harrison S., Minniti R., Wickes W., White J.M. (2010). Psychosocial Treatment for Methamphetamine Use Disorders: A Preliminary Randomized Controlled Trial of Cognitive Behavior Therapy and Acceptance and Commitment Therapy. Subst. Abus..

[59] Baker A., Lewin T., Reichler H., Clancy R., Carr V., Garrett R., Sly K., Devir H., Terry M. (2002). Motivational Interviewing among Psychiatric In-Patients with Substance Use Disorders. Acta Psychiatr. Scand..

[60] Smedslund G., Berg R.C., Hammerstrøm K.T., Steiro A., Leiknes K.A., Dahl H.M., Karlsen K. (2011). Motivational Interviewing for Substance Abuse. Campbell Syst. Rev..

[61] DiClemente C.C., Corno C.M., Graydon M.M., Wiprovnick A.E., Knoblach D.J. (2017). Motivational Interviewing, Enhancement, and Brief Interventions over the Last Decade: A Review of Reviews of Efficacy and Effectiveness. Psychol. Addict. Behav..

[62] Killeen T.K., Cassin S.E., Geller J., Brewerton T.D., Baker Dennis A. (2014). Motivational Interviewing in the Treatment of Substance Use Disorders, Addictions, and Eating Disorders. Eating Disorders, Addictions and Substance Use Disorders: Research, Clinical and Treatment Perspectives.

[63] Christie G.I.G., Cheetham A., Lubman D.I. (2020). Interventions for Alcohol and Drug Use Disorders in Young People: 10 Key Evidence-Based Approaches to Inform Service Delivery. Curr. Addict. Rep..

[64] Kumpfer K.L. (2014). Family-Based Interventions for the Prevention of Substance Abuse and Other Impulse Control Disorders in Girls. ISRN Addict..

[65] Roozen H.G., De Waart R., Van Der Kroft P. (2010). Community Reinforcement and Family Training: An Effective Option to Engage Treatment-Resistant Substance-Abusing Individuals in Treatment. Addiction.

[66] Donovan D.M., Ingalsbe M.H., Benbow J., Daley D.C. (2013). 12-Step Interventions and Mutual Support Programs for Substance Use Disorders: An Overview. Soc. Work. Public. Health.

[67] De Giorgi R., Cassar C., Loreto D’alò G., Ciabattini M., Minozzi S., Economou A., Tambelli R., Lucchese F., Saulle R., Amato L. (2018). Psychosocial Interventions in Stimulant Use Disorders: A Systematic Review and Qualitative Synthesis of Randomized Controlled Trials. Riv. Psichiatr..

[68] De Crescenzo F., Ciabattini M., D’Alò G.L., De Giorgi R., Del Giovane C., Cassar C., Janiri L., Clark N., Ostacher M.J., Cipriani A. (2018). Comparative Efficacy and Acceptability of Psychosocial Interventions for Individuals with Cocaine and Amphetamine Addiction: A Systematic Review and Network Meta-Analysis. PLoS Med..

